# Metabolomics as a Crucial Tool to Develop New Therapeutic Strategies for Neurodegenerative Diseases

**DOI:** 10.3390/metabo12090864

**Published:** 2022-09-14

**Authors:** Débora Lanznaster, Giulia Dingeo, Rayhanatou Altine Samey, Patrick Emond, Hélène Blasco

**Affiliations:** UMR1253, iBrain, INSERM, University of Tours, 37000 Tours, France

**Keywords:** metabolomics, neurodegenerative diseases, glutamate, therapy

## Abstract

Neurodegenerative diseases (NDs), such as Alzheimer’s (AD), Parkinson’s (PD), and amyotrophic lateral sclerosis (ALS), share common pathological mechanisms, including metabolism alterations. However, their specific neuronal cell types affected and molecular biomarkers suggest that there are both common and specific alterations regarding metabolite levels. In this review, we were interested in identifying metabolite alterations that have been reported in preclinical models of NDs and that have also been documented as altered in NDs patients. Such alterations could represent interesting targets for the development of targeted therapy. Importantly, the translation of such findings from preclinical to clinical studies is primordial for the study of possible therapeutic agents. We found that N-acetyl-aspartate (NAA), myo-inositol, and glutamate are commonly altered in the three NDs investigated here. We also found other metabolites commonly altered in both AD and PD. In this review, we discuss the studies reporting such alterations and the possible pathological mechanism underlying them. Finally, we discuss clinical trials that have attempted to develop treatments targeting such alterations. We conclude that the treatment combination of both common and differential alterations would increase the chances of patients having access to efficient treatments for each ND.

## 1. Introduction

Neurodegenerative diseases (NDs) are characterized by the progressive loss of neurons of specific types and from specific locations in the central nervous system (CNS). As NDs are primarily diseases of the elderly, the incidence of such diseases will also increase dramatically. Today, 30 million people are suffering from a neurodegenerative condition worldwide, and this number is predicted to reach more than 150 million by 2050—for Alzheimer’s disease alone, this number is expected to rise to 115 million people [1,2]. NDs are heterogeneous in their clinical presentations and underlying physiology. The three most common NDs are Alzheimer’s disease (AD), Parkinson’s disease (PD), and amyotrophic lateral sclerosis (ALS). They primarily affect hippocampal neurons, neurons from the substantia nigra pars compacta, and motor neurons from the motor cortex and spinal cord, respectively.

Even if much effort has been applied to understanding the pathological mechanisms behind neurodegeneration, it remains difficult to diagnose and treat such diseases, as symptoms usually appear several years after neuronal death has started. For example, for AD, studies have suggested that neurodegeneration starts 20–30 years before the onset of symptoms [3,4]. Moreover, ND diagnosis relies on clinical assessment, which can delay the definitive diagnosis even more—for ALS, it can take around 1 year [5]. Today, only palliative treatments are available for ND patients, as no cure has been found yet. Drugs on the market can only slightly decrease disease progression or symptoms and have a minor effect on improving survival [6]. Furthermore, they are usually accompanied by a number of side effects, due mostly to systemic effects rather than targeting the specific neuropathological alteration underlying the ND under treatment.

NDs share diverse pathological mechanisms, such as synaptic loss, neuroinflammation, glutamatergic toxicity, mitochondria dysfunction, oxidative stress, and protein aggregates mostly found in specific brain areas (for example, the hippocampus for AD, the substantia nigra pars compacta for PD, and the motor cortex and spinal cord for ALS) [6,7,8,9]. Furthermore, ND patients are known to present systemic alterations, such as hypermetabolism in ALS and insulin resistance in AD. Interestingly, several metabolomic studies have highlighted these systemic, metabolic alterations in diverse NDs [10,11]. Recent findings point to the application of metabolomic findings for the development of new therapeutic strategies. By identifying the pathways and metabolites that are altered in a patient or a subgroup of patients, we can envisage the “correction” or “compensation” of this altered pathway to stimulate and restore its normal function.

One of the major setbacks that prevents the development of efficient therapy to treat NDs is the use of non-appropriated preclinical models. Unfortunately, drugs that show beneficial effects in preclinical models often arrive in clinical trials and fail to show any improvement in patients [12]. Preclinical mouse models are useful for the identification of biomarkers for neurodegenerative diseases, but some effort has to be made to focus on alterations related to the pathological mechanisms behind each disease observed in patients. The discovery of appropriate biomarkers would help to improve patients’ management, allowing for early diagnosis and following the therapeutic efficacy of new treatments. In the present review, we aimed to identify metabolomic alterations found in patients with NDs that have also been reported in the preclinical models for AD, ALS, and PD. We analyzed these alterations to find (1) metabolomics disturbances common among AD, ALS, and PD and (2) specific disturbances that occur in each ND. This type of analysis allowed us to identify the metabolic alterations that are common to the three studied NDs and that may be related to the shared pathophysiology of NDs. Such knowledge can increase our understanding of neurodegenerative processes and improve patients’ management. We also discuss the few studies that have targeted such alterations in order to find a better treatment for these NDs.

## 2. Common and Specific Metabolite Alterations among AD, ALS, and PD

**Figure 1:**
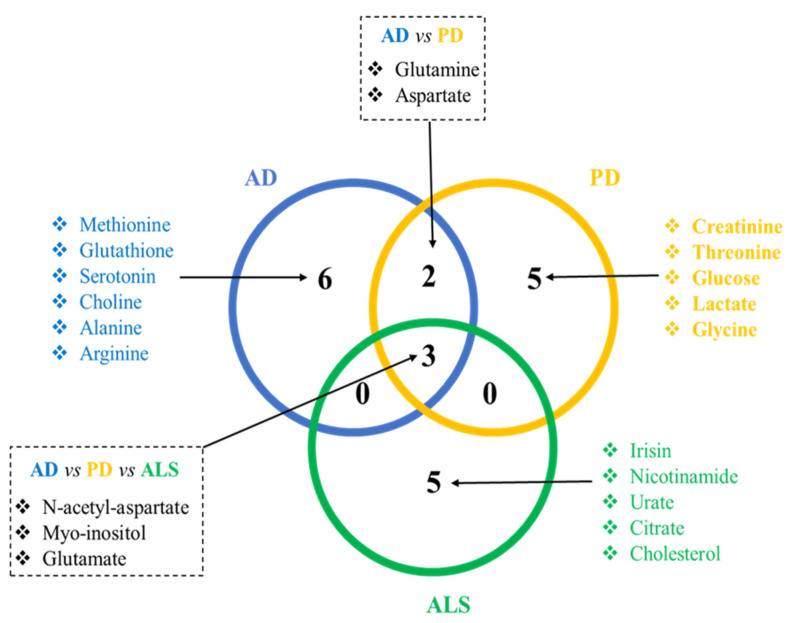
**Venn diagram showing the discriminant metabolites that are common and specific for each ND.** Discriminant metabolites were reported in preclinical models and in ND patients. This analysis shows that some metabolic alterations are common to the NDs discussed here, and disease-specific metabolic alterations are probably associated with the specific pathological mechanisms associated with each disease.

As mentioned before, NDs share common physiopathological mechanisms. As a consequence, it is expected that common metabolomics alterations can be found among patients with AD, ALS, and PD. Indeed, a meta-analysis from 2016 analyzed studies from humans and reported several metabolites that were suggested as common biomarkers for the three NDs, such as uric acid, choline, creatine, L-glutamine, alanine, creatinine, and N-acetyl-L-aspartate [10]. As each ND presents its own specificity (i.e., specific pathological mechanisms that target a specific subtype of neurons, specific proteins that aggregate and induce neurotoxicity for each disease), it is also logical to find alterations in metabolomics studies that are specific for each disease. In the present review, we analyzed original studies published until March 2021 that reported significant differences in metabolites in animal models of AD, PD, and ALS compared to their respective controls. Next, we searched for these alterations in studies performed in ND patients—regardless of the sense of the variation (i.e., increase or decrease) and analyzed matrices (brain tissue, CSF, or plasma). Regarding the number of studies that have published metabolite alterations in ND patients that were also described in preclinical studies, we retained 13 studies for ALS, 6 for AD, and 7 studies for PD. Some studies have reported more than one metabolite alteration, while other studies have reported the same metabolite alterations. The retained studies for each ND are presented in Table 1. We identified the metabolites altered in both settings (preclinical and clinical) and analyzed the sorted metabolites in a Venn diagram (Figure 1) to identify the common metabolites for AD, ALS, and PD. We also identified the metabolites that have been reported to be altered in preclinical and clinical studies for each disease.

In the next sections, we discuss these similarities, with emphasis on the pathological alterations behind the findings. We also discuss the metabolites sorted for each independent disease.

### 2.1. Common Metabolite Alterations to the Three Diseases: N-acetyl-aspartate, Myo-inositol, and Glutamate

When we searched for the common metabolite alterations among AD, ALS, and PD, we observed three common discriminant metabolites: N-acetyl-aspartate (NAA), myo-inositol, and glutamate. The studies discussed here are consistent with the hypothesis that these molecules may provide a marker for neuronal dysfunction. There is only one study that reported alterations in these three metabolites in AD patients. Starting from the hypothesis that reduced glutamate neurotransmission could contribute to cognitive damage in AD, this group carried out a pilot study to determine the activity of the neuronal tricarboxylic acid cycle. They enrolled 3 AD patients and 3 controls. The 6 subjects were examined with quantitative 1H and 13C MRS using a [1-13C] glucose infusion protocol. The team found out that in the patients, the levels of NAA/creatinine, [NAA], myo-inositol/NAA, and [myo-inositol]/[NAA] were significantly increased compared to the controls. The measures of the neuronal TCA cycle, glucose oxidation, and glutamate neurotransmission with magnetic resonance spectrometry (MRS) were significantly correlated with the measures of neuronal integrity [52].

#### 2.1.1. N-Acetyl-Aspartate (NAA)

N-acetyl-aspartate (NAA) is an abundant free amino acid present in a mammalian brain. It is found in neurons, and its concentration is correlated with neuronal mitochondrial function [53]. NAA is usually presented as a ratio to choline (Cho) or creatinine (Cr), reflecting the energy metabolism and degeneration, respectively, in MRS studies. It has been shown in the literature that NAA is significantly reduced in AD, PD, and ALS, as a probable reflection of the mitochondrial impairment and neurodegeneration observed in these three NDs. In this sense, NAA might be a potential biomarker of brain dysfunction that could contribute to the diagnostic process.

Regarding the data found in the literature concerning PD, different clinical trials have reported decreased NAA/Cho ratios in idiopathic PD patients compared to age-matched controls using MRS. These differences have been observed in brain areas associated with PD symptoms [54,55]. Early-stage PD is also associated with decreased levels of NAA/Cr in the globus pallidus (GP) and substantia nigra (SN), making it an interesting early biomarker for PD diagnosis [56]. Another clinical trial followed 13 patients with PD before and after bilateral subthalamic nucleus stimulation (STN-DBS), an effective treatment for advanced PD. The NAA/Cho and NAA/Cr ratios from the right and left globus pallidus, and left fronto-basal cortex were compared before the operation (patients were in the “off state”), and 3 months postoperatively (the stimulator was off). They showed that both the NAA/Cho and NAA/Cr ratios increased after STN-DBS, making them an interesting biomarker for the follow-up of treatment efficacy [57]. Intriguingly, the NAA levels were not associated with the occurrence of alpha-synuclein in a PD mouse model, reflecting that neurodegeneration occurs as a consequence of protein aggregation [58].

Regarding AD, a Chinese meta-analysis from 2015, comprising a total of 1519 healthy controls and 1282 AD patients, reported that the NAA levels in AD patients were significantly reduced in the posterior cingulate cortex (PC) and bilateral hippocampus, as was NAA/Cr in the posterior cingulate cortex [59]. A more recent meta-analysis confirmed the decrease in NAA (both as the metabolite alone and in the NAA/Cr ratio) in these areas as well as in the parietal white matter [60]. Interestingly, decreases in NAA/Cr in the inferior colliculi associated with a slow auditory response were reported in early-stage AD patients, making these non-invasive techniques an interesting tool to help AD diagnosis [61].

Decreased absolute NAA levels in ALS patients were reported by an Italian clinical trial [62], later on by a Canadian controlled clinical trial [63]. and more recently by a Japanese study [64] by means of MRS. More recently, a prospective multicenter study showed that NAA ratios in the motor cortex were related to disease progression and severity. Moreover, the NAA ratios were decreased in the prefrontal cortex only in cognitively impaired patients, making NAA a useful prognostic biomarker for ALS [65]. NAA levels could also be used in the clinic for follow-up treatment efficiency, as demonstrated in recent clinical trials [66].

#### 2.1.2. Myo-Inositol (mIns)

Myo-inositol (mIns) is one of the nine stereoisomers of inositol, a polyalcohol that belongs to the complex of vitamin B. It plays an important role in the morphogenesis and cytogenesis of cells, the synthesis of lipids, and cell membrane formation and is a precursor of second messengers and cell growth [67]. mIns is also considered a marker of neuronal integrity [68] and gliosis [69]. Glial cells may have a storage function for mIns, which can then be gradually passed on to the neurons, where it becomes a precursor in the phosphoinositide cycle [70].

In PD patients, a French study from 2016 reported that mIns levels were significantly lower in patients with parkinsonian syndromes in drug-off conditions than in healthy volunteers [71]. In 2019, a German study reported lower mIns levels in PD patients that were predictive of impaired gross and fine motor functions, primarily in the thalamus [72].

Increases in mIns were reported in AD patients’ posterior cingulate and in the parietal gray matter when compared to controls [59]. Another study showed an increase in the temporoparietal region that could be contributed to the differential diagnosis compared to frontotemporal dementia (FTD), which showed mIns increases in the frontal lobe [73]. mIns increases may also contribute to AD pathogenesis, since it was shown to inhibit catalase (an important antioxidant enzyme) and increase H_2_O_2_ levels, contributing to cell death [74].

Regarding ALS patients, one MRS study from 1993 found a significant increase in mIns in the precentral gyrus of ALS patients compared to healthy subjects [63]. mIns levels in the mesial prefrontal cortex of ALS-FTD were also found to be increased by 11% compared to the controls [75]. Moreover, mIns levels in the mesial prefrontal cortex were reported to be predictive of survival over an observation period of two years, but not at five years [76].

#### 2.1.3. Glutamate

Glutamate is an excitatory neurotransmitter in the mammalian central nervous system, and it plays a crucial role in memory, neuronal development, and synaptic plasticity [77]. However, its overstimulation is a well-known pathological mechanism of neurodegeneration called excitotoxicity [78]. Curiously, the data on glutamate levels in ND patients are very controversial, and their use in clinics as a biomarker is still far from being a reality.

A decrease in CSF glutamate levels in PD patients was first reported in 1997, followed by an increase in the levels of glutamine [28]. Since then, several studies have reported similar alterations. A meta-analysis concerning PD included 19 studies on the CSF concentrations of glutamate, demonstrating that glutamate was decreased in PD patients compared to age- and sex-matched controls [79]. The finding of decreased levels of glutamate in CSF is counter-intuitive. If glutamatergic neurons are hyperactive in ND, it might be expected that the levels of glutamate in the CSF should be increased. This might mean that the functional over-activity of the glutamatergic system is primarily due to an increase in the density or effector coupling of the glutamate receptors, an effect which would be predicted to lead to a compensatory decrease in the activity of the glutamate-releasing neurons, and thus, a decline in the extracellular and CSF concentrations of the amino acid.

Surprisingly, a meta-analysis from 2018, which included 35 studies on AD patients, with a total of 605 AD patients and 585 controls, showed that the CSF glutamate levels in the patients were not significantly different than those of the controls, rejecting the hypothesis of glutamate excitotoxicity and GABAergic resistance to neurodegeneration [80].

The data regarding ALS patients are conflicting. Several studies have reported increased glutamate levels in the blood and CSF using different techniques (MRI or LCMS, for example) [40,41,42,43,81,82]. Brain areas with reported increases in glutamate by MRS studies are the medulla [83], the supplementary motor area [64], and the left precentral gyrus [84]. However, other groups have reported decreased glutamate levels in the motor cortex [85]—while there are reports of no difference at all in the glutamate levels between ALS and the controls [86,87]. These results could partially explain the great heterogeneity found in ALS patients.

### 2.2. Common between PD and AD: Glutamine and Aspartate

#### 2.2.1. Glutamine

Glutamine stands out as a common altered metabolite between AD and PD. Glutamine is an amino acid that plays an important role in neurodegenerative diseases, and its concentration is regulated by the glutamate–glutamine cycle [88,89,90]. In the literature, it has already been demonstrated that glutamine synthetase, the astrocytic enzyme that converts glutamate to glutamine, plays an important role in neuroprotection [91,92,93].

Indeed, in AD, it has been shown that the observed neuronal loss was correlated to the deregulation in the glutamatergic system, and a dysfunction in this system can influence memory, cognition, and behavior. Decreased glutamine synthetase activity was identified in the postmortem brains of patients with AD [94,95,96,97]. Additionally, Aβ peptides were shown to directly inhibit glutamine synthetase activity in vitro [98]. Decreased glutamine synthetase activity in AD may be a part responsible for the increased level of glutamate and decreased level of glutamine observed in AD.

Regarding PD, even if no differences were found regarding the glutamine synthetase enzyme in PBMC from PD patients, [99], another study reported higher levels of glutamine in the plasma [100]. In the CSF, lower levels of glutamine were associated with akinesia [101]. These results provide further evidence that glutamate–glutamine metabolism is dysregulated in PD.

Because most studies have shown downregulation of the glutamine metabolism, which could contribute to the excitotoxicity induced by glutamate, modulating the activity of the glutamate synthetase in astrocytes could represent an interesting target for neuroprotection.

#### 2.2.2. Aspartate

Aspartate: The function, interaction, and disorders of aspartate in the pathophysiology of NDs are still poorly understood. In PD, there is a significantly higher concentration of aspartate in the midbrain, right cortex, right cerebellum, and right hypothalamus of the PD rat model compared to the control [30]. In AD, a study showed that aspartate concentrations remained unchanged in the gray matter between AD patients and the controls, while it was 2 times higher in the white matter in the AD patients [102]. In contrast, another study demonstrated that aspartate was significantly higher in the gray matter of AD patients [103]. These results raise questions about the involvement of aspartate in the modulation of cognitive and behavioral manifestations of AD and PD. In neurons, aspartate is converted to N-acetyl-aspartate (NAA) by the enzyme aspartate-N-acetyltransferase [104]. Considering that NAA is decreased in NDs, this could mean that the activity of the aspartate-N-acetyltransferase enzyme is decreased. However, the role of aspartate in the pathophysiology of these diseases deserves to be elucidated further by longitudinal studies on a larger scale.

### 2.3. Sorted Metabolites for AD: Alanine, Arginine, Methionine, Glutathione, Choline, and Serotonin

We found six metabolites reported in the literature as altered in AD patients and preclinical models. Regarding sorted amino acids, it was shown that alanine concentrations were increased in the white and gray matter of the brains of AD patients compared to the controls [105] while Lin et al. [106] demonstrated that there was a correlation between elevated alanine levels and behavioral symptoms.

In the case of arginine, arginase catalyzes the hydrolysis of arginine to form urea and ornithine, whose role is to contribute to the cellular detoxification of ammonia in the urea cycle, which protects the cells against ammonia toxicity [107]. Ornithine can be further metabolized to form putrescine, spermidine, and spermine polyamines, which are essential for normal cell growth and functioning, or via a separate pathway to form glutamate and γ-aminobutyric acid (GABA) [108]. Nitric oxide synthase uses arginine as a substrate to produce nitric oxide (NO) and citrulline [109]. There is also evidence to suggest that NO can directly modulate Aβ production and protect against increased Aβ [110]. In addition to its role as a substrate for enzymes, arginine is also a precursor for the biosynthesis of certain proteins, such as proline, creatine, and polyamines [108]. In this context, several studies have suggested the involvement of arginine metabolism in the pathogenesis of AD [111,112,113]. Indeed, it has been observed that alterations in the arginine levels and its metabolites in areas were strongly affected in AD, suggesting their possible contribution to neuropathology and cognitive disorders [114,115]. In addition, moderate decreases in the level of arginine have been detected in the CSF and plasma of patients with AD [116,117]. These studies indicate that arginine deprivation could be a critical pathogenic factor for AD, which ultimately leads to neuronal death and cognitive deficits. However, it remains to be determined at what stage of the disease process the arginine alterations begin to occur.

Methionine is a precursor of homocysteine, a neurotoxic sulfur amino acid intermediate in the methylation, and a precursor of glutathione, a tripeptide protective antioxidant agent [118,119]. The scientific literature has already shown that the disruption of the methionine metabolism has been linked with AD [120,121,122,123,124,125]. High methionine was also found to increase neuronal degeneration and impair short-term memory [126,127]. Chronic methionine administration also enhances the activation of microglia and inflammation and decreases neurogenesis in the hippocampus, which is characteristic of neurodegenerative diseases such as AD [122,124,128,129]. Glutathione is a major antioxidant that plays a crucial role in the antioxidant defense system and the maintenance of redox homeostasis in neurons [130]. Reductions in the levels of brain antioxidant glutathione have also been found to play a critical role in oxidative imbalance [131,132,133,134]. In addition, numerous scientific publications have found glutathione depletion in the brain area responsible for higher levels of cognition in AD and have demonstrated that this glutathione depletion aggravates oxidative damage in AD [135,136,137,138].

It has long been established that there is a decrease in choline concentrations in some cerebral regions of AD patients [139]. This decrease could be the result of increased choline flow in the methionine cycle and transmethylation reactions. This hypothesis can be supported by the results of Mahajan, Varma, Griswold, Blackshear, An, Oommen, Varma, Troncoso, Pletnikova, O’Brien, Hohman, Legido-Quigley, and Thambisetty [15], demonstrating overexpression of the choline dehydrogenase (CDHD) gene in AD. This mitochondrial enzyme oxidizes choline to betaine aldehyde in the methionine cycle [140]. These results suggest that increased use of choline in the transmethylation pathway by its conversion to betaine could reduce its availability for the synthesis of acetylcholine in the cholinergic neurons.

Serotonin is a neurotransmitter widely distributed in the central and peripheral nervous systems, mainly in the hippocampus, hypothalamus, and cortex. It is well known that serotonin dysfunction exists in AD patients and is manifested in the reduction of serotonin and its metabolites in the brains of elderly AD patients, the loss of serotonin neurons in the raphe nucleus, and decreased serotonin receptors in the cortex and hippocampus [141,142,143]. In addition, disruptions in serotonergic signaling have been reported to enhance amyloid-β pathology in in vitro, in vivo, and human clinical studies [144,145,146]. Previous literature suggested the mechanistic route for this influence is a result of serotonin receptor activation upregulating α-secretase activity, shifting the cleavage of the amyloid precursor protein away from the β- and γ- secretase route and reducing amyloid-β production [144].

### 2.4. Sorted Metabolites for PD: Creatinine, Threonine, Glycine, Glucose, and Lactate

Two amino acids were found in our analysis as differential for PD: glycine and threonine. Regarding L-threonine, its CSF levels in PD patients were higher compared to the controls, with a significantly lower CSF/plasma ratio [147]. Other studies further support the alterations in threonine and its role as a biomarker for PD [79,148,149]. Furthermore, it was shown that the metabolism of glycine and threonine was shown to be downregulated at the onset of alpha-synuclein aggregation in a mouse model of PD [26].

Glycine is an important inhibitory transmitter in the CNS [150]. In 1992, Iwasaki et al. directed a study with 20 patients with idiopathic PD (under levodopa treatment) and 20 controls. They found that the glycine levels were significantly increased in the plasma of patients with PD [151]. Several studies have reported that patients with PD also had elevated concentrations of glycine in the CSF [152,153,154].

Creatinine (Cr) is an endogenous compound produced primarily from creatine in muscle [155]. A study carried out in 2015 showed that nine metabolites—among them, creatinine, glucose, and lactate—had multivariate significant differences from the controls [25]. It was also shown that the Cr levels were already slightly lower one year before diagnosis compared to the controls [156]. Cr is the metabolic product of creatine. Due to its beneficial effect on mitochondria function, creatine was studied as a therapeutic agent in several cellular and animal models of PD, with interesting therapeutic results. However, when tested in clinical trials, no therapeutic effect was observed in PD patients [157].

Regarding glucose, it has been reported that patients with PD have a tolerance to glucose [158,159]. The connection could be therapy with levodopa [160] or the sharing of the same dysregulated pathways [161]. Interestingly, a clinical study showed that subthalamic nucleus–deep brain stimulation (STN-DBS) affected the glucose metabolism in PD patients [162]. It was also shown that the blood glucose level and the total area under the time curve (AUC) were significantly higher in PD subjects after an oral glucose tolerance test (OGTT), without differences in insulin levels compared to the controls [163]. Clinically, PD and diabetes are dissimilar, but they share genetic susceptibilities that put individuals at risk for both diseases [161]. A single nucleotide polymorphism in *AKT*, the encoding gene for AKT kinase that regulates cell survival and metabolism, increases the risk for both pathologies [164]. Interestingly, patients with Type 2 diabetes mellitus (T2DM) have a lower concentration of the protein DJ-1 in pancreatic islets, a protein codified by the PD-related *park7* gene [165].

One of the possible causes of lactate involvement in PD is that a number of genes associated with it are linked to mitochondrial activity [166,167]. An example of one of these genes is DJ-1/PARK7, mentioned above [168]. The deficiency of DJ-1 is associated with abnormal mitochondrial morphology and functions, increased sensitivity to oxidative stress, decreased mitochondrial membrane potential, and the opening of the mitochondrial permeability transition pore [169,170]. DJ-1 has been classified as a novel glyoxalase family [171]. Glyoxalases are enzymes that can transform 2-oxoaldehydes glyoxal and methylglyoxal into corresponding 2-hydroxy acids, glycolate, and D-lactate. Glyoxal and methylglyoxal covalently react with proteins or lipids to form advanced glycation end-products (AGEs), which are implicated in neurodegenerative diseases (PD included) [172,173]. Based on this hypothesis, German researchers showed that the products of DJ-1 (glycolate and D-lactate), regarding HeLa cells and *C. elegans*, were fundamental to maintaining the mitochondrial membrane potential. Moreover, they increased the in vitro survival of primary dopaminergic neurons from PD mice embryos. In addition, they found that the metabolites of the glyoxalases were components of a novel pathway that maintained high mitochondrial potential during cellular stress and that the production of glycolate and D-lactate was required to prevent the degeneration of dopaminergic neurons in the *substantia nigra* [168].

### 2.5. Sorted Metabolites for ALS: Irisin, Nicotinamide, Urate, Citrate, and Cholesterol

Irisin is a fragment of fibronectin type III domain-containing protein 5 (FNDC5/FRCP2/PeP), a type I membrane protein [174,175]. FNDC5′s C-terminal fragment is in the cytoplasm, while the N-terminal extracellular portion is proteolytically cleaved to produce irisin, which will be released in the circulation [176]. *FNDC5* is expressed in human muscle [177], and irisin is considered to be a myokine that leads to increased energy expenditure by stimulating the “browning” of white adipose tissue [178]. An animal study (MLC/SOD1^G93A^ mice) detected a significant decrease in irisin expression compared to the controls. This decrease could unbalance the muscle–nerve connection [46]. In patients, an Italian study from 2018 compared the levels of irisin between ALS patients and healthy subjects and found that all ALS patients presented higher irisin serum levels compared to the controls. Interestingly, when compared by subgroups of the patients, hyper-metabolic ALS patients had higher levels of irisin than the normo-metabolic ones [45].

Nicotinamide (or niacinamide) is the active form of vitamin B3. It is converted in the body starting from nicotinic acid (or niacin). It is essential for more than 200 enzymatic reactions to the NADH and NADPH coenzymes [179,180]. Sirtuins, or class III histone deacetylases, are NAD+ -dependent protein deacetylases [181], and studies have highlighted the implication of sirtuins in ALS, from animal models [182,183,184] to postmortem patients [185,186]. Nicotinamide adenine dinucleotide (NAD+) diminution is correlated to many neurological pathologies, causing the accumulation of neurotoxic proteins in the nervous central system [187]. Recently, a study determined the effect of increasing NAD+ availability in ALS mouse models. They used two approaches; the first one was the ablation of an NAD+- consuming enzyme (CD38), and the second one was supplementation with a bioavailable NAD+ precursor (nicotinamide riboside—NR; 400 mg/kg/day until their death). In their study, NR-supplementation delayed motor neuron degeneration, decreased markers of neuroinflammation in the spinal cord, appeared to modify muscle metabolism, and modestly increased the survival of hSOD1^G93A^ mice [184].

Citrate is produced in the Krebs cycle (or TCA cycle) from the aldol condensation of oxaloacetate and acetyl-CoA [188]. There is an interconnection between citrate and several cellular processes, for example, protein modification and the shift between carbohydrate and fatty acid metabolism [189]. Citrate synthase (CS) activity in ALS patient platelets was shown to be increased [190]. Although a recent study showed no differences in CS activity in the muscle form of early-stage ALS patients, they demonstrated several pathological changes in the energetic metabolism of muscle [191]. These results suggest dysfunctions that may lead to increased energy requirements in the muscles and brains of ALS patients [49].

Uric acid (or urate, at the physiological plasma pH) is the main catabolic product of purine bases (adenosine and guanosine) that derive from the degradation of nucleic acids (typically DNA, RNA, and food-derived) [192]. It accounts for most of the antioxidant capacity in human plasma [193,194]. Due to this property, it is protective against neurodegeneration [195]. It has long been shown that ALS patients have urate levels in plasma or serum lower than healthy controls [196,197,198,199]. With this in mind, a clinical trial for dexpramipexole evaluated the urate concentration in serum to determine if the urate concentration could predict ALS progression. They found that BMI had a positive correlation with urate, while age was negatively correlated with urate levels in men. After adjusting for the BMI, there was not a significant trend of improved survival or slower disease progression with increasing urate levels [200]. However, another study reported that decreased uric acid levels were correlated to the rate of disease progression (ALSFRS-R decline) [198].

Cholesterol is transported in the blood by lipoproteins. It has a role as a precursor of steroid hormones, vitamin D, and bile acid [201]. It was reported that high plasma levels of cholesterol could be neuroprotective for ALS [51], and it might be associated with the improvement of survival [202]. In 2014, a Swedish study found a positive correlation between cholesterol levels and survival in ALS [203]. A later study found that ALS patients with higher triglyceride levels had longer survival times compared to patients with lower levels [204].

## 3. Correcting Metabolite Alterations by Targeted Therapy

Once the metabolite alterations were identified, we could envisage that targeting such alterations—by trying to compensate for such imbalances—could be promising for the development of more efficient therapy for NDs. However, despite all metabolite alterations reported so far by different studies, few clinical trials have evaluated the therapeutic potential of reestablishing or regulating metabolite levels in ND patients. Even fewer have followed these alterations as outcome biomarkers for verifying treatment efficacy. Here, we review the few studies that have tested the therapeutic effect of targeting these metabolite alterations.

Glutamatergic excitotoxicity represents the most targeted common alteration in NDs, as several anti-glutamatergic drugs have been tested, but only a few have been approved for treatment. This is the case for memantine for AD, riluzole for ALS, and lately, amantadine for PD. However, their therapeutic effect, followed by numerous side effects, remains a challenge—possibly due to the conundrum of the increase/decrease in glutamate discussed before. The potential therapeutic effect of anti-glutamatergic drugs may benefit from novel techniques for the measurement of cerebral glutamates, such as glutamate-weighted chemical exchange saturation transfer (gluCEST) MRI, which is currently used in clinics to assess glutamatergic overactivity in epileptic patients [205].

For the case of arginine in AD, it was shown that the administration of arginine in the alimentation of patients with senile dementia increased cognitive function by about 40% [206], while an epidemiological study indicated that the intake of dietary arginine is inversely correlated with AD morbidity [207]. Arginine was also shown to significantly reduce levels of total serum cholesterol and low-density lipoproteins [208], so decreasing the levels of arginine could be one of the causes or consequences of lipid dysregulation in patients with AD. Furthermore, considering that cholesterol is also dysregulated in ALS, arginine could present a potential therapeutic effect in ALS models and patients.

It was also hypothesized that the upregulation of arginase activity and the resulting arginine and NO deficiency in brain areas, characterized by excessive amyloid deposition, contribute to the clinical manifestation of AD [209]. As a result, the bioavailability of arginine is a regulatory factor for the synthesis of several proteins essential for neuronal survival. In order to improve its availability, the combination of pharmacological agents targeting enzymes in the arginine metabolism could represent a beneficial approach for treating AD.

Glutathione (GSH) deficiency is linked to AD, and prior studies have shown that GSH deficiency can be corrected by supplementing its precursors, glycine and cysteine (provided as N-acetylcysteine, NAC), with their combination, termed GlyNAC. An ongoing randomized clinical trial (NCT04740580) is evaluating the effect of GlyNAC versus alanine (placebo) supplementation during a period of 24 weeks in patients with AD, and will measure changes in cognition, GSH concentrations, oxidative stress, brain glucose uptake, brain inflammation, and insulin resistance. The results are expected in May 2025.

PD patients were shown to present dysregulation in their glucose metabolism [210], and recent epidemiology studies have shown that T2DM not only increased the risk for PD, but is also associated with PD clinical severity [211]. In this sense, several anti-diabetic drugs tested in animal models and PD patients have shown interesting therapeutic effects. Accordingly, a phase II ongoing trial is testing the safety of a synthetic analog of human glucagon-like peptide-1 (GLP-1), Exenatide (NCT04305002). The study is expected to be completed by October 2022. Another ongoing clinical trial is testing the safety and tolerability of intranasal insulin in PD patients, with the results expected by January 2023 (NCT04251585).

NAD+ supplementation has been shown to improve the growth of neural stem cells (NSCs) and neuronal precursor cells (NPCs) (Ref). The treatment of a mouse model of ALS (SOD1-G39A) with 20 mg/mL of nicotinamide riboside for 50 days regulated mitochondrial proteostasis and improved neurogenesis through the activation of the mitochondrial unfolded protein response [212]. Considering ALS patients, one single-center, double-blind placebo-controlled randomized controlled trial recruited 32 patients, and for 4 months, they evaluated the differences between EH301 (composed of 1-(β-D-Ribofuranosyl) nicotinamide chloride and 3,5-Dimethoxy-4′-hydroxy-trans-stilbene) and placebo treatments. After the 4 months, they found that participants that received EH301 demonstrated significant amelioration in the ALSFRS-R score, pulmonary function, muscular strength, and in the skeletal muscle/fat weight ratio. Moreover, EH301 displayed a significantly slower rate of progression of the disease compared to the placebo [213].

**Figure 2:**
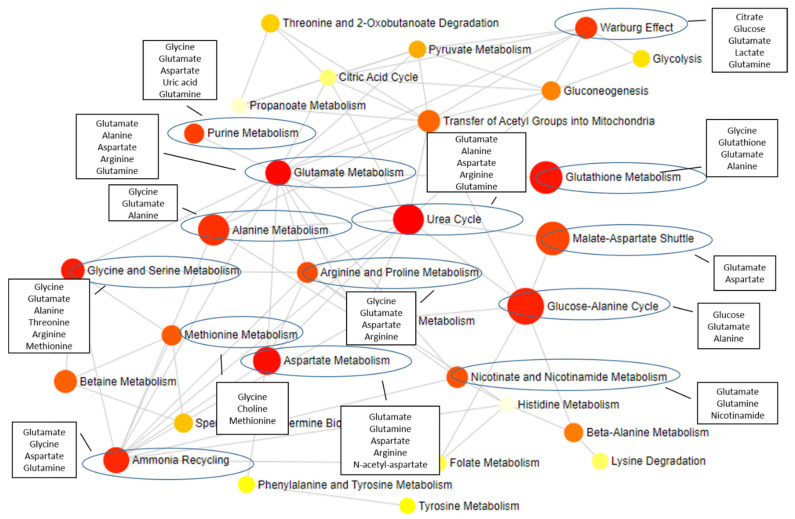
Metabolites described in this review and the impacted pathways. The metabolites identified as altered in NDs (in black rectangles) participate in several metabolic pathways, with a major impact on pathways highlighted with a blue circle. The grey lines represent the connections between different pathways. Figure designed using MetaboAnalyst 5.0 [214].

The identification of differentially and commonly altered metabolites for each ND opens up the perspective for treatment improvement; we can envisage, for example, a combined therapy that targets metabolite alterations common among NDs (such as N-acetyl-aspartate, myo-inositol, and glutamate) but also differential alterations for each disease. By targeting both alterations at the same time, it would be possible to “correct” multiple pathological pathways alterations underlying each disease. A combined therapy based on metabolome changes has the potential to improve therapeutic efficacy with fewer side effects [82]. Since most of the metabolic pathways communicate with each other (Figure 2), therapy targeting metabolism alterations have the potential to affect multiple pathways at once.

Furthermore, by starting the treatment scheme targeting these common alterations even before definite diagnosis, patients would benefit from early intervention, improving the chances of observing a beneficial effect. This is especially important for NDs, since diagnosis can be delayed for several months, as is the case of ALS. Moreover, targeting the alterations found in preclinical models would increase the chances of observing similar beneficial effects of the same treatment in patients.

## 4. Conclusions

In this review, we focused our attention on metabolites described to be altered both in preclinical models and in studies performed on ND patients. In this sense, our findings are limited by the reported studies since some metabolites are known pathological alterations described for patients, but have not been reported by preclinical studies. For example, choline levels were already reported to be altered in PD patients, while creatinine levels are known to be altered in ALS patients due to muscle loss. Furthermore, alterations in the glutathione levels were shown for all three NDs discussed here, as well as alterations in energetic metabolites, such as glucose, lactate, and cholesterol. These alterations were not found and discussed in our study because preclinical studies have not shown the same alterations or simply because no study has investigated such alterations in preclinical models. With this approach in mind, we suggest that (1) preclinical studies should investigate if known alterations in patients could also be found in the preclinical model, which would improve the model’s characterization and validity, and (2) preclinical studies should investigate the pathological mechanism behind the described alterations in metabolites, improving our knowledge on the pathophysiology of NDs.

Neurodegenerative diseases, such as AD, PD, and ALS share common pathological mechanisms but present specificities, for example, the neuronal type affected and the protein biomarkers in degenerated cells. Treatments that target mechanisms common to these diseases would improve the number of ND patients that would benefit from a therapeutic option. After decades of failed clinical trials in search of new and better therapeutic options to treat NDs, the development of therapies targeting more than one pathological mechanism is an utmost need. In this case, by targeting alterations described both in pre-clinical models and patients, we would improve our chances of finding better treatments that, once moved to clinical trials, would finally find their way to clinical practice.

## Figures and Tables

**Table 1:** Studies reporting metabolomics alterations found in patients with NDs that were also reported in preclinical models.

Alterations in Patients	Preclinical Studies
**AD**	
Methionine: increased in CSF [13]	[14]
Glutathione: increased in CSF [13]; increased in brain tissue [15]	[14]
Serotonin: decreased in CSF [16]; increased in CSF [13]	[17]
Choline: decreased in brain tissue [15]	[18]
N-acetyl-aspartate: decreased in brain tissue [15,19]; increased in brain tissue [20]	[14,21,22]
Glutamine: increased in CSF [23]; increased in brain tissue [20]	[17,18]
Alanine: increased in brain tissue [19]	[18]
Myo-inositol: increased in brain tissue [19]	[18]
Glutamate: increased in brain tissue [20]	[21,22]
Arginine: increased in brain tissue [20]	[14,17,21]
Aspartate: decreased in brain tissue [20]	[18]
**PD**	
Creatinine: decreased in CSF [24]; increased in CSF [25]	[26]
Threonine: increased in CSF [27]	[26]
Glutamine: increased in CSF [27,28]; increased in SN [29]	[26,30]
Glucose: increased in CSF [25,31]	[32]
Lactate: increased in CSF [25]	[26]
Glutamate: decreased in CSF [28,33]; increased in SN [29]	[30]
Aspartate: decreased in CSF [33]	[26,30]
Glycine: decreased in CFS [33]	[26]
N-acetyl-aspartate: decreased in SN [29]	[34]
Myo-inositol: decreased in SN [29]	[26]
**ALS**	
N-acetyl-aspartate: decreased in motor cortex [35,36]	[37,38]
Myo-inositol: increased in motor cortex [39]	[37,38]
Glutamate: increased in blood [40,41]; increased in CSF [42,43]	[38,44]
Irisin: increased in serum [45]	[46]
Nicotinamide: decreased systemic and in CSF [47]	[47]
Urate: decreased in plasma [48]	[37]
Citrate: increased in CSF [49]	[37]
Cholesterol: increased in plasma [50,51]	[37]

CSF: cerebrospinal fluid; SN: substantia nigra.
